# Metabiotics: One Step ahead of Probiotics; an Insight into Mechanisms Involved in Anticancerous Effect in Colorectal Cancer

**DOI:** 10.3389/fmicb.2016.01940

**Published:** 2016-12-02

**Authors:** Mridul Sharma, Geeta Shukla

**Affiliations:** Department of Microbiology, Panjab UniversityChandigarh, India

**Keywords:** metabiotics, metabolites, short chain fatty acids, probiotics, colorectal cancer

## Abstract

Colorectal cancer is closely associated with environment, diet and lifestyle. Normally it is treated with surgery, radiotherapy or chemotherapy but increasing systemic toxicity, resistance and recurrence is prompting scientists to devise new potent and safer alternate prophylactic or therapeutic strategies. Among these, probiotics, prebiotics, synbiotics, and metabiotics are being considered as the promising candidates. Metabiotics or probiotic derived factors can optimize various physiological functions of the host and offer an additional advantage to be utilized even in immunosuppressed individuals. Interestingly, anti colon cancer potential of probiotic strains has been attributable to metabiotics that have epigenetic, antimutagenic, immunomodulatory, apoptotic, and antimetastatic effects. Thus, it’s time to move one step further to utilize metabiotics more smartly by avoiding the risks associated with probiotics even in certain normal/or immuno compromised host. Here, an attempt is made to provide insight into the adverse effects associated with probiotics and beneficial aspects of metabiotics with main emphasis on the modulatory mechanisms involved in colon cancer.

## Introduction

Cancer refers to a heterogeneous collection of neoplastic cells evolving in microenvironments with complex ecologies (Aktipis and Nesse, 2013). Colorectal cancer (CRC) is the third most common cancer, affecting mainly colon and rectum in both men and women and is the second leading cause of cancer-associated mortality (Cancer Facts and Figures, 2016). CRC affects more than 1 million people annually, accounting for about 500,000 deaths worldwide and its incidence is increasing at an alarming rate. Further, CRC has been predicted to affect at least half of the western population by the age of 70 due to consumption of red or processed meat, alcoholic beverages, less fruits and vegetables as well as lack of physical activity (Jemal et al., 2010). Recently, World Health Organization has predicted about 27 million new cases of cancer, 17 million deaths and about 75 million people will be affected with CRC by 2030 (Kumar et al., 2015).

The etiology of CRC is complex comprising of a well-defined series of histological changes paralleled with mutational activation of oncogenes and inactivation of tumor suppressor genes regulated by a multifactorial interplay between diet, environment, carcinogenic chemicals, and mutagens (Sankpal et al., 2012; Raman et al., 2013). It has been proposed that sporadic cancer arises due to mutant low-penetrance genes which interact extensively with environmental factors (Birt and Phillips, 2014). The most common driving mutations of colon cancer affect adenomatous polyposis coli (APC), rat sarcoma family of genes (*ras*), phosphatidyl inositol-3-kinase (PI3K), and transforming growth factor (TGFβ) (Vogelstein et al., 2013).

CRC can be treated either by surgery, radiotherapy or chemotherapy predominantly with 5-fluorouracil and oxaliplatin depending on the progressional stage but due to systemic toxicity, resistance to chemotherapy and recurrence of cancer, alternate interventions are needed. Therefore, life style and dietary interventions such as exercise, fiber rich foods and fermented food products have been gaining a lot of interest by scientists round the globe for preventing colon cancer with main focus on probiotics. Probiotics are “Live microorganisms that, when administered in adequate amounts, confer a health benefit on the host” as per International Scientific Association for Probiotics and Prebiotics (Colin et al., 2014).

It is widely acknowledged that susceptibility as well as progression of colon cancer is determined by gene-environment interactions and microbial communities constituting micro environment of the gut. In 400 BC Hippocrates stated ‘Death sits in the bowel,’ implying the importance of gut microbiome in human health and Elie Metchnikoff too linked the longevity in Bulgarian peasants with consumption of fermented dairy products. The gut microbiome is metabolically so active that it is regarded as a complete organ which dynamically participates in various physiological functions and has 10 times more cells and 100 times more genes than human body (Nicholson et al., 2012). Colon cancer risk is influenced to a great degree by the balance between microbial production of health-promoting metabolites such as lactic acid, short chain fatty acids (SCFAs), linoleic acid, some glycoproteins/peptides and potentially carcinogenic metabolites such as amines and secondary bile acids. All these beneficial bioactive substances associated with probiotics are referred as either metabiotics, postbiotics, biogenics, or simply metabolites/CFS (Cell free supernatants). The term “metabiotics” refers to the structural components of probiotic microorganisms and/or their metabolites and/or signaling molecules with a determined chemical structure that can optimize host-specific physiological functions, regulatory, metabolic and/or behavior reactions connected with the activity of host indigenous microbiota (Shenderov, 2013). Among these, SCFAs are the most studied being a source of energy for colonocytes and modulator of various metabolic activities.

No doubt probiotic bacteria with antimutagenic and/or antigenotoxic activities, have been found to exert generalized prophylactic effect against colon cancer but SCFAs have been thought to be more selective in targeting cancer cells and regulating the expression of oncogenes and tumor suppressor genes by epigenetic mechanisms (Cousin et al., 2012; Verma and Shukla, 2014, 2015; Papadimitriou et al., 2015). Besides these, it has been reported that some of the gut bacteria, e.g., *Streptococcus gallolyticus, Bacteroides fragilis*, *Fusobacterium* have been implicated in the genesis of CRC. More specifically, the translocation of *Streptococcus gallolyticus* to blood stream has been linked to development of inflammation and colon tumor as well as toxin produced by *Bacteroides fragilis* has been shown to cause genetic alterations due to upregulation of *myc* and NFκB expression leading to CRC (Sears, 2009; Wu et al., 2009; Boleij and Tjalsma, 2013). Furthermore, Ahn et al. (2013) and Kostic et al. (2013) have also reported *Fusobacterium* to be the more dominant bacteria in colon adenomas. Although, it is very well-evident that probiotic microorganisms have health benefits, yet may cause opportunistic infections, increase the incidence of allergic sensitization and autoimmune disorders, alter the micro ecological balance, modify gene expression, transfer antibiotic resistance and virulence genes, lead to disorders in epigenome and genome integrity, induce chromosomal DNA damage and activate signaling pathways associated with cancer and other chronic diseases in certain healthy or in immunocompromised and high risk individuals, thereby limiting their use (Snydman, 2008; Gratz et al., 2010). To overcome such adverse effects associated with probiotics, metabiotics are preferred due to their known chemical structure, effective dose, safety assurance and longer shelf-life (Shenderov, 2013).

### Implications of Probiotics

Probiotics may be highly beneficial to the host as it has been described that they can maintain epithelial integrity, compete for adhesion and nutrition with pathogens, stimulate cell mediated immunity, IgA production and gut associated lymphoid tissue. Additionally, probiotics detoxify carcinogens, reduce serum cholesterol level, alleviate lactose intolerance, produce active metabolites including organic acids, bacteriocin, H_2_O_2_ and enhance the production of vitamins (Kechagia et al., 2013). Moreover, probiotics are also being used either as prophylactic or therapeutic agent for various diseases such as antibiotic induced or infectious diarrhea, ulcerative colitis, Crohn’s disease and irritable bowel syndrome (Verna and Lucak, 2010). Despite having a history of safe use, introducing live microbes in the human body could be a potential threat especially to immunocompromised and genetically predisposed individuals and is a matter of concern (Pan et al., 2014). Scientists have reported that an infant and a child without any underlying GIT disease or immunocompromised status suffered from bacteremia upon lactobacilli supplementation (Cabana et al., 2006). In addition, some medical reports have also highlighted that lactic acid bacteria and even bifidobacteria have been associated with human opportunistic infections such as infective endocarditis, sepsis, bacteremia, pneumonia, abdominal abscesses, peritonitis, meningitis, urological infections, rheumatic vascular diseases in immune compromised individuals and patients with allergic sensitization and autoimmune disorders (Ohishi et al., 2010; Berer et al., 2011). Though incidence of such cases may be lower, yet depends very much on probiotic species and strain specificity. Probiotic strains have also been found to increase platelet aggregation and aggravate hemolytic uremic syndrome; (Yazdankhah et al., 2009; Van Reenen and Dicks, 2011) and are potential source of toxic metabolites like biogenic amines in certain individuals (Bourdichon et al., 2012). It should not be ignored that some silent genes of probiotic bacteria may be induced by host cell signals during passage through intestinal tract, leading to undesirable effects (Di Caro et al., 2005). Probiotics have also been reported to transfer genetic information including antibiotic resistance to commensals or pathogens due to the presence of antibiotic resistance plasmids leading to genomic and epigenomic alterations (Shenderov, 2011).

*Enterococcus faecium*, having a long history of probiotic use in preventing antibiotic-associated diarrhea, may act as opportunistic pathogen owing to the presence of potential reservoir of antibiotic resistance and virulence genes (Dirienzo, 2013). A recurrent septicemia and central nervous system deterioration has also been observed in an immunocompromised patient with spores of the probiotic strains of *Bacillus subtilis* EG-RN and EG-CM (Fijan, 2014). Some studies have also revealed increased bacterial translocation leading to mortality upon *Lactobacillus delbrueckii* UFV-H2b20 and *Bifidobacterium lactis* Bb12 supplementation in mice with 1,2-dimethyl hydrazine (DMH)-induced injuries (Liboredo et al., 2010). Additionally, some genotoxic effects have also been linked to probiotic strain *Escherichia coli* Nissle 1917 possessing a set of genes (pks island) responsible for the induction of double-strand breaks in host cell DNA (Cuevas-Ramos et al., 2010).

It’s ironical that live microbes could potentially be risky in immuno compromised patients where probiotic applications are often considered, thereby emphasizing the careful examination and application of probiotics which in turn depends upon species and strains of probiotics and host status. Moreover, traditional way of administering probiotics may not be able to produce required concentration of the metabolites to produce desired effect at the target sites *in vivo*. Therefore, to enhance *in situ* production of beneficial metabolites, the administration of either probiotic or specific prebiotic may be useful or these multifunctional metabolites may be given instead of probiotics as such but needs to be thoroughly investigated before implementation.

### Physiological Effects of Metabiotics

**FIGURE 1:**
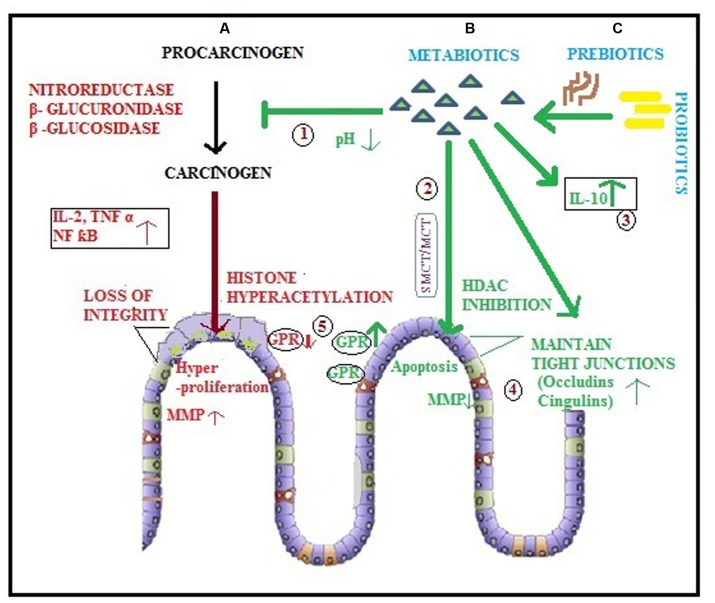
**Diagrammatic representation of colorectal cancer and various modulatory potentials of metabiotics.** **(A)** Procarcinogens are converted to carcinogens by enzymes leading to initiation of CRC which is accelerated by various factors such as inflammatory cytokines, genetic and epigenetic alterations (histone hyperacetylation) resulting into hyper proliferation of colonocytes that leads to metastasis due to lack of intestinal integrity and MMP production; **(B)** Metabiotics (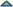) derived directly by probiotics (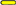) or **(C)** produced as a result of prebiotic (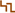) utilization by probiotic (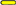) may: (1) inhibit the conversion of procarcinogen to carcinogen by creating a low pH environment in colonic lumen and hindering the synthesis as well as activity of harmful enzymes; (2) act as HDAC inhibitors via SMCT/MCT transporters leading to enhanced apoptosis of cancerous cells; (3) modulate inflammation by augmenting the level of anti-inflammatory cytokines (IL-10); (4) maintain intestinal integrity by ameliorating the expression of tight junction proteins (occludins, cingulins) and inhibit MMP to impede metastasis of cancer cells; (5) enhance the expression of GPRs, the cell surface receptors.

Probiotics colonize, multiply and produce variety of bioactive substances “metabiotics,” accounting for their beneficial effects in gastrointestinal tract (GIT) diseases. These metabolites produced by probiotics help in maintaining homeostasis in the gut and enhance the growth of friendly bacteria that inhibit the conversion of procarcinogens into carcinogens by decreasing harmful enzyme levels such as nitroreductase, β-glucuronidase and β-glucosidase (Verma and Shukla, 2013; **Figure 1** ‘1’). SCFAs also induce chemopreventive enzymes glutathione *S* transferase and Glutathione transferase pi (Scharlau et al., 2009; Johnson et al., 2012) and impart genetic stability to the colon cells (Clarke et al., 2012). Butyrate produced by fermentation of high amylose starch was reported to reduce the overall oxidative stress in gut and may also activate different procarcinogen metabolizing enzymes to aid in colon cancer prevention (Treptow-van et al., 1999). Butyrate acts as the preferred source of energy for colonocytes and has anti inflammatory and anticancerous properties, acetate percolates to peripheral tissues and could be metabolized in systemic areas (muscle) and generates ATP while propionate is transported to the liver via portal circulation (Hijova and Chmelarova, 2007). Acetate, a multifunctional SCFA plays significant role in regulating normal epithelial cell division, ileal motility, and colonic blood circulation (Hong et al., 2005). It was notable that cancer cell state was shifted from apoptosis to necrosis in an acidic extracellular pH created by exposure to SCFAs produced by probiotic *Propionibacteria freudenreichii* (Lan et al., 2007). Liong (2008) have shown that *Bifidobacterium* produced metabolites might interfere with the conversion of azoxymethane to its active carcinogenic form and subsequently lowered colon cancer risk. Interestingly, proteins derived from probiotic LGG, namely p75 and p40 have been reported to maintain gut homeostasis by regulating signaling mechanisms (Yan et al., 2007).

Short chain fatty acids stimulate overall health of the gut by improving mineral absorption, increasing mucosal weight, blood circulation and gut motility, thus preventing colitis which may gradually lead to cancer. Low pH environment created by SCFAs inhibits 7-α-Dehydroxylase and also renders free bile acid less soluble, hindering the generation of secondary bile acids (Lupton, 2000). Toxic metabolites produced in the human gut such as nitrates and oxides in response to inflammation have also been known to influence microbial populations and hence their metabolites formation (Bultman, 2014). Since, intestinal tract is constantly exposed to potentially harmful substances and toxic metabolites produced by them, it requires balancing by protective metabolites produced by the probiotics suggesting that an optimum balance between various metabolites ensures a healthy gut.

### Metabolites Lead to Epigenetic Alterations

The term “epigenetics” implies modification of the expression of certain genes induced by environmental factors without changing their DNA sequences, such as histone modifications (methylation, phosphorylation, deacetylation), DNA methylation, chromatin remodeling as well as mechanisms mediated by non-coding RNA molecules. Both initiation and progression of colon cancer include breach in epigenetic regulation of cell growth rate, differentiation and apoptosis which are influenced to a great degree by environmental factors (Coppedè, 2014). The epigenetic alterations paralleled with genetic mutations may lead to transformation of normal colonic mucosa to colorectal carcinoma. Histone deacetylases, a class of critical enzymes cause post-translational modifications of histone proteins leading to altered chromatin structure, influencing the binding of transcription factors and silencing tumor suppressor genes (Dokmanovic and Marks, 2005; Bolden et al., 2006). Further, it has been documented that overexpression of histone deacetylase is linked with severity of various cancers such as 62% expression in adenomas and 82% in colorectal carcinomas compared with 53% in normal human tissue (Ashktorab et al., 2009). Interestingly, these epigenetic changes are potentially reversible paving way for the discovery of a new class of drugs called “epigenetic drugs” or histone deacetylase inhibitors (HDACi) such as SCFAs (butyrate, acetate, propionate, and valerate) produced by good bacteria, the Probiotics (Licciardi et al., 2010; Kim et al., 2014; **Figure 1** ‘2’). These HDACi relax the chromatin giving access to transcription factors to activate the key target genes involved in anti cancerous effect as well as modulates the immune response by generating T regulatory cells (Arpaia et al., 2013; Park et al., 2015). The supernatant of human fecal slurry and pectin has been reported to have potent HDAC in inhibitory effects on colon cancer, due to production of butyrate (Waldecker et al., 2008) but the epigenetic effect of butyrate varies with type of dietary lipid intake and more pronounced effect was observed with fish oil compared with corn oil (Crim et al., 2008). Louis and Flint (2009) have reported *Faecalibacterium prausnitzii* and *Eubacterium rectal* to be the major butyrate producers in the gut. Furthermore, butyrate has been found to have potential to differentiate between cancer cells and normal cells in order to exert epigenetic effects and inhibits growth of colon cancer cells mainly by de-repressing epigenetically silenced genes such as p21 and the anti-apoptotic protein Bcl-2 but also promotes the growth of normal cells by regulating these genes (Zhong et al., 2014). Butyrate, the most studied SCFA, has been attributed in induction of apoptosis in colon cancer cells due to its ability to convert procaspase 3 to active caspase 3 (Medina et al., 1997). Furthermore, it has been found to induce both cell cycle arrest as well as terminal differentiation in HT-29 cells owing to the down regulation of cell cycle regulator CB1 mRNA expression and induction of cell cycle inhibitor p21 (Archer et al., 1998; Hinnebusch et al., 2002). To conclude, SCFAs offer a new strategy to fight colon cancer by acting as HDACi in order to modify all major functions such as induction of apoptosis, cell cycle arrest and differentiation. Since aberrant gene transcription due to epigenetic alterations is often the underlying cause of the onset of chronic inflammation and cancer, thus the epigenetic regulation by metabolites produced by probiotic microorganisms (Kumar et al., 2013) emerges as an explanation to the various anticancerous effects and suggests metabiotics as an alternative strategy for both cancer prevention and therapy or butyrate producers in the gut could be the target for enhancing *in situ* production of butyrate and its epigenetic benefits.

### Antimutagenic Properties Associated with Metabiotics

Antimutagenic potential of probiotics has primarily been attributed to binding of live bacteria with mutagens but now there is increasing evidence that even cell free supernatants might either scavenge the reactive carcinogen intermediates or influence the ability of carcinogen activating/deactivating enzymes (Treptow-van et al., 1999; Wollowski et al., 2001). Supernatants of probiotic cultures supplemented with prebiotics were reported to substantially reduce the genotoxicity of human fecal slurry (Burns and Rowland, 2004). Similarly, metabolites produced in soymilk fermented by mixed culture of *Streptococcus thermophilus, Lactobacillus acidophilus, Bifidobacterium infantis, Bifidobacterium longum* have also been found to exhibit high antimutagenicity against mutagen 3, 2-dimethyl-4-amino-biphenyl (Hsieh and Chou, 2006). Further, it was observed that colon cells treated with supernatant of inulin fermentation by lactic acid bacteria elevated Glutathione *S*-transferase-pi [(GST)- pi] activity, a chemopreventive enzyme against mutagens (Scharlau et al., 2009). The enhanced antimutagenic activity of cell free supernatant of *Lactobacillus plantarum* KLAB21 was attributed to three secretory glycoproteins 16, 11, and 14 kD (Rhee and Park, 2001). In addition, exopolysaccharides (EPSs) produced by *Lactobacillus plantarum* 301102 have also been shown to inactivate tryptophan pyrolysate-1, another known mutagen (Tsuda et al., 2008). Nadathur et al. (1995) prepared acetone extracts of yogurt and showed that active metabolites responsible for antigenotoxic activity in the extract vary with type of carcinogen as were found to be different for methylnitronitrosoguanidine (MNNG) and 3,2-dimethyl-4 aminobiphenyl (DMAB). In another interesting study, acetone extracts prepared from either non-fermented milk/fermented milk or *L. acidophilus* grown in MRS broth have been found to exhibit variable antigenotoxic activity as these metabolites prevented DNA damage in colonocytes to different degrees after MNNG treatment. These metabolites have been found to exert more profound antigenotoxic effect compared with cellular components of LAB like peptidoglycan or cytoplasmic fractions (Wollowski, 1998). However, mutagen binding potential of probiotics (lactobacilli and bifidobacteria) has been found to be associated with cellular components such as peptidoglycans and polysaccharides but the antimutagenic activity very much depends upon the growth phase, cell number of bacterial strain and mutagen type (Raman et al., 2013). Butyrate binds irreversibly to mutagen and is responsible for the antimutagenic potentials exhibited by probiotic microorganisms, lactobacilli and bifidobacteria (Stein et al., 1996; Lankaputhra and Shah, 1998). Further, butyrate enhances the production of (GST) – pi in colon cancer cells and has also been found to inhibit the genotoxic activity of nitrosamides and hydrogen peroxide production in human colon cells as well as in experimental model (Wollowski et al., 2001; Clarke et al., 2012). In addition to butyrate; acetate, another SCFA has also been documented to have antimutagenic properties (Christophersen et al., 2013). Taken together, antimutagenic potential of metabiotics has been linked with butyrate, acetate, some glycoproteins, peptidoglycans, and polysaccharides. It can be suggested that the metabiotics may exhibit antimutagenicity by various mechanisms such as irreversible binding to mutagens, inhibiting carcinogen activating enzymes, inducing chemopreventive enzymes, preventing DNA damage and offers them unique opportunity to be nominated as effective prophylactic agents against colorectal cancer.

### Immunomodulatory Potential

Inflammation and cancer are complementary to each other and the correlation between these was first observed by Virchow more than a century ago by observing the presence of leukocytes in neoplastic tissues (Francescone et al., 2014). About 20–25% of all cancers occur as a result of chronic inflammation due to its involvement both in tumor initiation and progression, e.g., inflammatory bowel disease leads to colon cancer, hepatitis to hepatocellular carcinoma and *H. pylori*-induced gastritis to gastric cancer (Grivennikov et al., 2010). Chronic and uncontrolled inflammation facilitates cancer development either due to epigenetic alterations, genomic instability, enhanced proliferation, invasiveness or resistance to apoptosis (Kundu and Surh, 2012). In fact, tumor itself can recruit pro-inflammatory cytokines, immune cells and growth factors in its microenvironment to enhance cancer development and create metastatic niche for secondary growth resulting into “Tumor elicited inflammation” (Coussens et al., 2013). Interestingly, tumor expression of oncogene k-ras upregulates the pro-inflammatory cytokine IL-8, resulting in immune cell infiltration, enlarged tumor and enhanced angiogenesis in colon cancer (Sparmann and Bar-Sagi, 2004). Moreover, damaged or leaky epithelial junctions in colon cancer too encourage vigorous inflammatory response that in turn accelerates tumor initiation and progression (Grivennikov et al., 2012).

Notably, degree of mucosal inflammation has been correlated with levels, type and proportion of various SCFAs. These metabiotics play important role in optimizing immune response generated by intestinal epithelial cells in order to prevent chronic inflammation. SCFAs maintain a balance by inhibiting inflammatory cytokines such as IL-2, IL-6, and TNF-α as well as stimulating the anti inflammatory cytokine IL-10 (Zhong et al., 2014; **Figure 1** ‘3’). SCFAs are known to regulate immune function possibly by monitoring neutrophils, antigen presenting cells, T-cell differentiation and histone deacetylase inhibition. In addition, SCFAs may also interact with G-protein-coupled receptors in the gut and affect inflammatory signaling by regulating chemotaxis of neutrophils and other immune cells such as dendritic cells and macrophages (Kimura et al., 2011; Vinolo et al., 2011).

In addition to SCFAs, various bio active factors derived from probiotics are EPSs, peptidoglycan, single layer proteins, lipoteichoic acids, conjugated linoleic acids and peptides that have also been found to regulate immune system (Matsuguchi et al., 2003; Lahtinen, 2012). Zakostelska et al. (2011) emphasized that even heat killed probiotics have equally effective anti inflammatory properties as live probiotics and suggested to adopt former as the safer option for treating chronic gastrointestinal inflammation. In nutshell, it can be stated that the efficacy of various metabiotics in modulating inflammation has been established using cell lines, murine models and even human trials mainly by upregulating the synthesis of antiinflammatory cytokines and down regulating the inflammatory mediators (**Table 1**).

**Table 1:** Immunomodulatory potential of metabiotics in different clinical and experimental studies.

Study	Reference
Intra-rectal infusion of SCFAs (80 mM sodium acetate, 30 mM sodium propionate, 40 mM sodium butyrate) twice a day up to 6 weeks in 12 patients suffering from ulcerative colitis led to notable amelioration of inflammatory responses.	Breuer et al., 1991
Treatment of patients suffering from ulcerative colitis with twice daily SCFA enemas (sodium acetate 80 mM, sodium propionate 30 mM, and sodium butyrate 40 mM) were helpful against colitis.	Vernia et al., 1995
Caco-2 cells treated with butyrate inhibited IL-1β-induced IL-8 mRNA expression and suppressed NFκB by inhibiting both its nuclear translocation as well as DNA binding and induction of IκB-β.	Wu et al., 1999
Metabolites produced by *Streptococcus thermophilus and Bifidobacterium breve* inhibited TNF-α secretion from lipopolysaccharide treated peripheral blood mononuclear cells or the THP-1 cell line.	Menard et al., 2004
Intestinal epithelial cells grown with conditioned media containing VSL#3-produced conjugated linoleic acids (CLA) enhanced PPARγ and CD36 expression by suppressing TNF-α and MCP-1 colonic expression in comparison to VSL#3 or linoleic acids alone.	Ewaschuk et al., 2006
The proteins p40 and p75 obtained from *LGG* activated protein kinase B and inhibited TNF-α.	Yan et al., 2007
The supernatant of *Faecalibacterium prausnitzii* DSM 17677 suppressed IL-1β-induced NFκB activation.	Sokol et al., 2008
Immunomodulation and anti colon cancer effect exhibited by *Lactobacillus casei* Shirota was attributed to a polysaccharide–peptidoglycan complex.	Matsumoto et al., 2008
A QS system related peptide produced by *B. animalis lactis* BB-12 enhanced the expression of c-myc and IL-6.	Mitsuma et al., 2008
*L. reuteri* secreted factors decreased NFκB p65 nuclear translocation by suppressing IκBα ubiquitination and modulated MAPK signaling.	Iyer et al., 2008
A small, heat stable and water-soluble anti inflammatory factor produced by *Saccharomyces boulardii* prevented the activation of NFκB reporter gene and IκBα degradation in THP-1 cells, leading to inhibition of p65 nuclear translocation and NFκB DNA binding.	Sougioultzis et al., 2006
An exopolysaccharide PSA, a cell wall component of *Bacteroides fragilis* reproduced the potent anti inflammatory effects as that of parent bacteria both *in vitro* and *in vivo*.	Round and Mazmanian, 2010
Bacterial extracts of *LGG* and *B. adolescentis* given to rats significantly activated macrophages to enhance the production of TNF-α and nitric oxide.	Lee et al., 2008; Bertkova et al., 2010; Switzer et al., 2011
Short chain fatty acids regulated chemotaxis of neutrophils dependent on GPR43 activation and other immune cells such as dendritic cells and macrophages.	Vinolo et al., 2011
The purified soluble protein p40 from *Lactobacillus rhamnosus* GG exerts anti-inflammatory effects in murine colitis model.	Yan et al., 2011
Furanosyl borate diester produced by *Escherichia coli Nissle*1917 induced the production of anti inflammatory cytokines by influencing the gene expression of *luxS*.	Jacobi et al., 2012
Conjugated leinoleic acids suppress TNF-α and MCP-1 colonic expression and reproduce the effects of VSL#3 administration in mice with Disodium sulfate colitis.	Bassaganya-Riera et al., 2012a,b
Butyrate treatment improved mucosal health of colitis murine model by restoring cytokine balance, normal crypt length and reducing the severity of inflammation.	Vieira et al., 2012
Oral administration of butyrate controlled T cell-induced colitis in lymphocyte deficient mice and ameliorated inflammation in mice models for ulcerative colitis.	Vieira et al., 2012; Furusawa et al., 2013
Various SLPs on the surface of *Propionibacterium freudenreichii* ITGP20 contribute to the induction of IL-10 and IL-6.	Le Marechal et al., 2014


### Antiproliferative and Apoptotic effects

Both *in vitro* and *in vivo* studies have demonstrated the significant role of bioactive components in cell free supernatants of probiotic strains, e.g., SCFAs (mainly butyrate) in direct inhibition of colon cancer cell growth by various mechanisms (**Table 2**). The most striking feature of butyrate is that it can differentiate phenotypically between normal and cancer cells as it induces differentiation, apoptosis and inhibits angiogenesis in cancerous cells but regulates the proliferation of normal cells (Davis and Milner, 2009).

**Table 2:** Anti proliferative and apoptotic effect of butyrate in colon cancer.

Study	Reference
Butyrate treatment reduces the colony forming ability of cancer cells as observed on soft agar along with induction of alkaline phosphatase in colon cancer cell lines.	Topping and Clifton, 2001
Butyrate modulates apoptosis even in genetically damaged cells both via extrinsic as well as intrinsic pathways mainly by upregulation of the proapoptotic protein BAK, caspase-3-mediated cleavage of poly-(ADP-ribose) polymerase (PARP) and downregulation of neuropilin-1, the key apoptotic and angiogenesis regulator of tumor cells.	Ruemmele et al., 2003; Yu et al., 2010
The administration of butyrate producing bacteria, *Butyrivibrio fibrisolvens* MDT-1 to experimental model of CRC resulted in a significant decline in ACF and β glucuronidase levels.	Fotiadis et al., 2008
Butyrate reduces the development of colon cancer by reducing the expression of vital genes, cyclin D1 and c-*myc*.	Maier et al., 2009
Anti cancerous potential of butyrate has been due to its ability to suppress miRNAs which are overexpressed in colon cancer. Altered expression of at least 44 miRNAs in HCT-116 cells as well as increased expression of p21 has been assessed due to the suppression of miR-106b on treatment with butyrate.	Hu et al., 2011
Hyperactivation of WNT/beta-catenin signaling pathway constitutes the major mechanism of butyrate to induce apoptosis in colon cancer cells.	Zeng et al., 2014
Combination of sodium butyrate together with a polyphenol, epigallocatechingallate (EGCG) proved to have synergistic effect against colon cancer as it induced significant degree of apoptosis, decreased colony forming capability of cancer cells by 80% and cell cycle arrest in different colon cancer cell lines (RKO, HCT-116 and HT-29). In addition to these, the expression of p21 and p53 were upregulated while HDAC and the caspase inhibitory protein, survivin were downregulated.	Saldanha et al., 2014
Butyrate treatment led to cell injury due to decreased adhesion and increased granulation of HCT-116 colon cancer cell lines.	Kazemi sefat et al., 2015


In addition to butyrate other SCFAs, propionate and acetate have also been reported to enhance apoptosis by affecting the mitochondrial trans-membrane potential, generating ROS, caspase-3-processing and causing condensation of nuclear chromatin in colon cancer cells (Jan et al., 2002; Lan et al., 2007; Hosseini et al., 2011). These SCFAs (propionate and acetate) are however required in higher concentrations to induce apoptosis and show different metabolic effects compared with butyrate (Topping and Clifton, 2001). It has been suggested that propionate also inhibits cancer other than in the gut as its being easily transported to liver via portal circulation (Bindels et al., 2012). Surprisingly, butyrate and propionate have also been found to induce autophagy rather than apoptosis in colon cancer cells due to some mitochondrial defects, revealing the reason behind mixed results observed with SCFA treatment. Further, this could result into the development of resistance in colon cancer cells suggesting the use of autophagic inhibitors to enhance the apoptotic effect of SCFAs (Tang et al., 2011a; Adom and Nie, 2013).

Apart from SCFAs, medium chain fatty acids such as capric, caprylic, and caproic acids have also been reported to be cytotoxic against colon cancer cell lines (Narayanan et al., 2015). Additionaly; long chain fatty acids (CLA) have been found to possess potent anticancerous effects. A commercially available probiotic mixture VSL#3 and few strains of *Bifidobacterium* have been shown to produce CLA, a potent inducer of apoptosis, COX-2 inhibitor and activator of peroxisome proliferator-activated receptor gamma (PPAR γ) in colon cancer and have also been able to mitigate intestinal tumorigenesis in *Apc^-^/^+^* mice (Davis and Milner, 2009; Urbanska et al., 2009; Uccello et al., 2012).

Kim et al. (2008) reported that CFS of *Bifidobacterium adolescentis* SPM0212 was more potent in inhibiting growth of colon cancer cells than whole cells or heat killed cells. CFS of *Enterococcus lactis* IW5 was also found to induce apoptosis in various cancer cell lines including Caco-2 and HT-29 (Nami et al., 2015). Similarly, CFS of probiotics (*Lactobacillus plantarum* A7 and LGG) were more effective in inhibiting cancer cell growth mainly due to organic acids produced by them compared with *E. coli* supernatant (Sadeghi-Aliabadi et al., 2014). In addition to organic acids, EPS (released EPSs, cell bound EPSs) of *Lactobacillus rhamnosus* ATCC 9595 also showed antiproliferative effect against HT-29 cells (Kim et al., 2006). Recently, it was highlighted that the upregulation of early apoptosis gene markers *cfos* and *cjun* constitutes the major mechanism involved in cytotoxicity of cell free supernatants of probiotic cultures toward colon cancer HT-29 and HCT116 cells (Shyu et al., 2014). Further, it has been documented that supernatants of *Lactobacillus delbrueckii* were able to induce apoptosis through intrinsic pathway and downregulation of Bcl-2 along with arrest of colon cancer cells in G1 phase (Wan et al., 2014). Conditioned medium of *Bacillus polyfermenticus* (BPCM) effectively suppressed the growth of colon cancer cells (HT-29, DLD-1, and Caco-2) and reduced their colony formation on soft agar due to decreased cyclin D1, ErbB2, and ErbB3 [epidermal growth factor receptors (EGFRs)] expression and inhibited tumor growth in mice because of heat stable bacterial proteins (Ma et al., 2010). LGG homogenate and cytoplasm extracts have been found to reduce the percentage of colon cancer cell viability to almost 55% in DLD-1 colon cancer cell lines; while heat killed LGG reduced the viability of HT-29 and Caco-2 to 62.7 and 73% respectively (Choi et al., 2006; Orlando et al., 2009). Very recently, it was reported that metabolites produced by new potential probiotic strains (*Pediococcus pentosaceus* and *Weissella confusa*) were cytotoxic against Caco-2 cell lines (Sevda et al., 2015). Based on these inferences, it is reasonable to postulate that metabolites produced by probiotics possess antiproliferative activity due to their ability to induce differentiation, arrest cell cycle and upregulate the proapoptotic mechanisms in cancer cells which in turn depends upon the phenotypic state of cells, probiotic strain and the active component involved.

### Intestinal Integrity and Metastasis Inhibition

Leaky gut syndrome (LGS) refers to increase in intestinal permeability and occurs either due to irregular expression of certain tight junction proteins or epithelial barrier dysfunction. LGS plays important role in promoting inflammation and cancer development by encouraging tissue invasion and metastasis. It could be both cause and effect of gastrointestinal ailments like inflammatory bowel disease and even colon cancer (Wang et al., 2005). Probiotics contribute to intestinal homeostasis due to the production of metabiotics (such as SCFAs and polyphosphates) which exert protective gut barrier effects by regulating induction, maintenance and assembly of tight junction proteins, production of mucin and/or gastrointestinal peptides employed in barrier function (Rao and Samak, 2013).

Butyrate, the major probiotic metabolite in CRC not only acts as HDAC inhibitor and regulator of apoptosis but also promotes intestinal epithelial barrier integrity by regulating the expression of various tight junction proteins such as occludins and cingulin (Bordin et al., 2004; Peng et al., 2009; **Figure 1** ‘4’). Caco-2 cells when treated with cell-free supernatant of *B. lactis-*420 showed improved integrity of tight junctions (Ewaschuk et al., 2008; Putaala et al., 2008). A decrease in colonic permeability was also recorded by oral administration of bioactive factors from *B. infantis* in murine colitis model (Segawa et al., 2011). Metabolites produced by *B. infantis* Y1 increased ZO-1 and occludin expression in T cells (Rao and Samak, 2013). It has been observed that surface components of probiotic microorganisms such as pilus, lipoteichoic acids, and mucus binding proteins interact directly with both epithelial and immune cells of the host to maintain gut barrier homeostasis. In addition, Patel et al. (2012) reported an interesting observation that even heat-killed LGG regulated intestinal barrier in mice with abnormal gut. Recently p40, a soluble protein produced by LGG has been found to enhance the chemical barrier by activating EGFR which in turn stimulates goblet cells to produce mucin (Wang et al., 2014). Both p40 and p75 protect not only the epithelial tight junctions against oxidative stress through MAP kinase signaling pathways but also inhibit the redistribution of tight junction proteins induced by hydrogen peroxide (Seth et al., 2008). Cell-free conditioned media of LGG has also been observed to induce the production of cytoprotective heat shock proteins (hsp25, hsp72) by intestinal epithelial cells which promote tight junctions (Thomas and Versalovic, 2010). LGG supernatant is capable of reversing the epithelial barrier dysfunction induced by alcohol, both *in vitro* as well as *in vivo* and have been found to regulate levels of claudin-1, ITF, P-gp and cathelin-related antimicrobial peptide to re- establish intestinal barrier functions (Wang et al., 2011, 2012). Competence and sporulation factor (CSF), a quorum-sensing peptide produced by *Bacillus subtilis* JH642 has the potential to induce hsp25, hsp27, and hsp72 in intestinal epithelial cells (Fujiya et al., 2007). It has also been observed that proteins derived from *Clostridium butyricum* CGMCC0313-1 regulate the intestinal barrier function by enhancing the expression of protein A20 in HT-29 cells (Song et al., 2012).

Metastasis is the major cause of mortality in cancer patients. Metastasis or excessive proliferation and invasion of cancerous cells occurs due to degradation of extracellular matrix by enzyme matrix metalloproteinases (MMPs) and MMP-9 levels have been significantly correlated with the stage of colon cancer (Baker and Leaper, 2002; Leeman et al., 2003; Mook et al., 2004). Colorectal cancer metastasis occurs due to loss of tight junction proteins such as occludin, ZO-1 and clodulin-4 (Kaihara et al., 2003; Tobioka et al., 2004; Ohtani et al., 2009). Escamilla et al. (2012) have demonstrated that metabolites produced by *L. casei* and LGG potentially interfere with metastatic process in colorectal cancer by decreasing MMP-9 activity and increasing the levels of ZO-1 in HCT-116 cells. Butyrate hindered the activity of MMPs due to activation of tissue inhibitor matrix metalloproteinase (TIM) 1- and 2 (Adom and Nie, 2013). Another study observed that supernatants of *Lactobacillus delbrueckii* ameliorated cell invasion in colon cancer cells by decreasing MMP-9 activity (Wan et al., 2014). Further, it was revealed that fermented products of synbiotic (inulin and oligofructose with LGG and/or *Bifidobacterium lactis* BB12) inhibited cell invasion both *in vitro* and in experimentally induced colon carcinogenesis in rats (Pool-Zobel, 2005; Pool-Zobel and Sauer, 2007). In nutshell, it can be stated that metabolites produced by probiotics offer a safe and effective tool for combating colon cancer by their ability to maintain the intestinal integrity; regulating tight junctions and to inhibit metastasis by decreasing MMP activity.

### Receptors and Transporters Associated with Biological Actions of Metabiotics

Continuous extensive research on the interaction of bacterial metabolites especially SCFAs with host has advanced toward the molecular approach of butyrate and other SCFAs toward their modulatory mechanisms in colon, both extracellularly (via activating specific receptors) and intracellularly (via transportation mediated by specialized transporters) (Ganapathy et al., 2013). SCFAs synthesized by friendly bacteria in the lumen of colon could be absorbed passively or carried via transporters like SMCT1/SLC5a8 and MCT1/SLC16a1 into colonocytes. SMCT1 and MCT1 are sodium-coupled monocarboxylate transporter and H^+^-coupled transporter respectively. Despite the presence of SMCT1 along entire length of the large intestine, MCT1 found in apical membrane of colonocytes accounts for the major transport of SCFAs and could be transported either by apical or basolateral membrane of colonocytes. MCT1 is also expressed on the apical membrane of dendritic cells, kidney cells, and even brain cells and is downregulated during early adenoma formation accompanied with enhanced expression of the high affinity glucose transporter, GLUT1 and reduced expression of the low affinity glucose transporter GLUT2 (Lambert et al., 2002).

Undissociated SCFAs can passively diffuse across the apical membrane of epithelial cells lining but due to acidic intestinal pH; most of the SCFAs are dissociated and have to be transported actively via transporters (Sellin, 1999). Scientists have proposed three fundamental mechanisms for the active transport of SCFAs including SCFA-HCO3^-^ exchange, electroneutral SCFA anion cotransport with cations mediated by MCT1 and electrogenic sodium-dependent transport carried out by SMCT1 (Besten et al., 2013). Gonçalves et al. (2011) have reported that butyrate is transported faster from apical membrane of colonocytes via SMCT-1 compared with acetate and propionate and essential for histone deacetylase inhibition (Li et al., 2003; Gopal et al., 2004; Miyauchi et al., 2004). SCFAs not only enter colonocytes but also the immune cells via SMCT1 thereby hindering the inflammatory signal by blocking the generation of dendritic cells from bone marrow stem cells (Singh et al., 2010).

Short chain fatty acids are not fully consumed by colonocytes but have to be transported actively across their basolateral membrane via cation-SCFA anion symport or SCFA -HCO3^-^ antiport and is proposed to be mediated by MCT4 (*SLC16A3*) and MCT5 (*SLC16A4*) (Gill et al., 2005). SCFAs can also enter other organs such as liver and muscle via portal circulation. The transporters for butyrate and propionate across sinusoidal membrane of liver cells have been identified as the organic anion transporters OAT7 and OAT2 respectively (Shin et al., 2007; Islam et al., 2008).

Receptors present on different cells play important role in pathophysiology of various diseases. GPR 109A and GPR43/free fatty acid receptor (FFA2) are cell- surface G-protein-coupled receptors expressed on the colonic epithelium, adipose tissue, and immune cells which are activated by SCFAs (Blad et al., 2012; **Figure 1** ‘5’). GPR41/free fatty acid receptor 3 (FFA3) is another receptor found on colonocytes, adipocytes, spleen, lymph nodes, bone marrow, renal smooth muscle cells, enteric neuronal cells, and pancreatic cells (Xiong et al., 2004; Kim et al., 2014). Butyrate and niacin (Vitamin B3) are the known potential agonists for GPR109A while acetate and propionate are for GPR 43 receptors (Bindels et al., 2013). FFA2 has higher affinity for both acetate and propionate and is present primarily in immune cells whereas FFA3 has greater affinity for butyrate and propionate but has highest expression in adipose tissue (Brown et al., 2003; Le Poul et al., 2003; Nilsson et al., 2003). However, FFA2 suppresses inflammation in healthy colon as reduced expression of GPR43 has been found both at the primary site of tumor as well as the metastatic sites in CRC. Further, cancer cells with increased expression of GPR43 were found to be more susceptible for apoptosis and cell cycle arrest caused by SCFAs (Tang et al., 2011b). Acetate, has been able to impart protection to germ-free mice from dextran sulfate sodium (DSS) induced colitis by acting as a GPR43 Singh et al. (2014). The expression of neuropilin-1 (receptor for VEGF) has been inversely correlated with levels of SCFAs in colon and has been shown to be reduced by butyrate in colon cancer cell lines (Yu Danny et al., 2011).

GPR109A and SLC5A8 (SMCT1) both aid significantly in enhancing apoptosis induction potential of butyrate which implies both extracellular as well as intracellular regulation (Andoh et al., 2003; Thangaraju et al., 2008). GPR109A is responsible for enabling macrophages and dendritic cells to differentiate T regulatory cells and induce production of anti- inflammatory cytokines as evident by the ability of induction of expression of IL-18 in colonic epithelium by butyrate and niacin in WT mice (possessing GPR109A) but inability to do so in case of *Niacr1-/-* mice (lacking GPR109A) (Singh et al., 2014). This study has shown that animals deficient in GPR109A are more susceptible to both inflammation-induced and mutated *Apc* driven colon carcinogenesis. Cresci et al. (2010) reported that the levels of SLC5A8 mRNA and GPR109A mRNA in colon were markedly reduced in germ-free mice but restored with recolonization of the gut microflora by maintaining conventional conditions for 3–4 weeks as gut bacteria play important role in the expression of both receptors and transporters of SCFAs. Moreover, optimal dietary fiber intake increases the concentration of SCFAs in the gut lumen leading to entry of butyrate and other HDAC inhibitors into the cell even without SLC5A8 via direct diffusion or by other low affinity monocarboxylate transporters such as SLC16A1. But SLC5A8 becomes indispensable in case of low dietary fiber intake, as SCFAs in low concentrations will not be able to diffuse or enter the cell via low affinity transporters. However, GPR109A and GPR43 are essential for extracellular functions of SCFAs in the presence of both, optimal as well as low dietary fiber intake.

The immune modulating activity of either probiotic or metabiotic is thought to be due to binding of SCFAs to GPRs, that helps in recruitment of immune cells, activation of effector T cells and production of cytokines and chemokines (Sina et al., 2009; Smith et al., 2013). SCFAs have been extensively involved in the modulation of the expression of both receptors and transporters associated with various cells which hold paramount importance in pathogenesis of CRC. Thus, it can be stated that the interactions between SCFAs and these receptors/transporters need to be explored to reveal the molecular mechanism of action of metabiotics.

## Future Directions

Although, it may take a while to unravel and utilize the remarkable attributes associated with metabiotics against colon cancer but it seems quite possible due to increasingly overwhelming studies emerging in this direction such as commercialized metabiotics, ‘Hylak Forte’ (Lactic acid and SCFAs) and ‘Zakofalk’ (Inulin and butyrate) derived from multiple probiotics to promote general health and to treat moderate inflammatory conditions respectively (Belousova et al., 2005; Roda et al., 2007). Moreover, probiotic strains should be screened for specific potential metabiotics in their cell free supernatants as there is much ground to be covered yet. However, delivery of metabiotics to the right place (i.e., distal colon) however remains a challenge in CRC which could be met by devising new strategies of targeted delivery. The fact that metabiotics affect gene expression even at translational level by altering miRNAs has added another dimension to their mechanistic study but warrants detailed investigation. Though considerable amount of *in vitro* work regarding the undeniable role of metabiotics in colon cancer has been accomplished but intensive *in vivo* studies followed by placebo controlled, double blind clinical trials need to be performed.

## Conclusion

Probiotics are known to exert various health benefits such as immunomodulation, inactivation of carcinogens and maintenance of gut integrity but the present review argues the notion that live probiotic strains are essential for the beneficial effects associated with them in colon cancer. Moreover, adverse effects associated with probiotics even in certain normal/or immunosupressed individuals limit their use. Thus, attempts are being made to have alternative safer and effective biointerventions such as metabiotics and to enhance their *in situ* production by selectively optimizing the growth conditions for beneficial probiotics. Moreover, metabiotics, the multifunctional metabolites produced by probiotics have been found to possess remarkable antimutagenic, antiinflammatory, antiproliferative and even antimetastatic potentials attributed to their epigenetic effects in one or other way, and may target CRC at different stages. This information suggests that the administration of metabiotics may attenuate both the adverse effects as well as worry of maintaining the viability of probiotics. Thus, metabiotics independently or in conjunction with other approaches could be considered as a potent prophylactic/or therapeutic modulator for colon cancer or other diseases in the post-antibiotic era.

## Author Contributions

Collected and compiled information: MS; wrote the paper: MS, GS; edited: GS.

## Conflict of Interest Statement

The authors declare that the research was conducted in the absence of any commercial or financial relationships that could be construed as a potential conflict of interest.

The reviewers FT and MM and handling Editor declared their shared affiliation and the handling Editor states that the process nevertheless met the standards of a fair and objective review.
